# The lncRNA PHAROH augments MYC mRNA levels in insulin-producing cells by counteracting TIA1-induced MYC silencing

**DOI:** 10.1038/s41598-026-65911-9

**Published:** 2026-08-18

**Authors:** Tongjian Zhao, Nils Welsh

**Affiliations:** Department of Medical Cell Biology, Uppsala University, Box 571, Uppsala, SE-751 23 Sweden

**Keywords:** Beta cell, MYC, lncRNA, T-cell intracellular antigen 1, RNA-binding protein, Endocrinology, Endocrine system and metabolic diseases

## Abstract

Type 1 and type 2 diabetes are characterized by beta cell dysfunction and insulin deficiency, and the appropriate turnover of MYC expression appears to control the beta-cell fate. Therefore, it is important to better understand the mechanism that controls MYC expression. Hence, the current study aimed to elucidate the interactions of the long non-coding RNA molecule PHAROH at the interface of MYC mRNA and the RNA-binding protein TIA1. For this purpose, we studied MYC mRNA levels in glucagon-producing alpha-TC1-6 cells, insulin-producing MIN6 cells, and in human pancreatic islets. We observed that in insulin-producing cells, but not in alpha cells, MYC mRNA levels were increased in response to stress (proinflammatory cytokines or palmitate + high glucose). Levels of PHAROH were also increased in insulin-producing cells, but not in glucagon-producing cells. To evaluate whether the increased PHAROH levels participated in the induction of MYC mRNA, we treated MIN6 cells with synthetic antisense LNA GapmeR oligonucleotides (ASO) designed to target PHAROH. We observed that the PHAROH ASO 1 increased both PHAROH and MYC mRNA expression. MYC protein levels were also increased by PHAROH ASO 1, both in the absence and presence of pro-inflammatory cytokines. Using an RNA immunoprecipitation strategy, we observed that PHAROH ASO 1 decreased MYC mRNA binding to TIA1, and instead increased PHAROH binding to TIA1. It is concluded that beta cells increase their MYC expression during stress and that this occurs via increased PHAROH-mediated sequestration of TIA1 and subsequent release of MYC mRNA from TIA1-induced inhibition.

**Supplementary Information:**

The online version contains supplementary material available at 10.1038/s41598-026-65911-9.

## Introduction

Diabetes is primarily characterized by chronic hyperglycemia, caused by an absolute or relative lack of insulin^1,2^. In type 2 diabetes, the insulin-producing beta cells are negatively affected by metabolic overload, pro-inflammatory cytokines, oxidative stress and other factors that reduce beta cell function^3^. The transcription factor MYC, also known as c-Myc, plays a pivotal role in regulating the expression of genes associated with cell proliferation, apoptosis and metabolism^4^. In beta cells, MYC is necessary for neonatal and adaptive cell replication^5,6^, and it appears that MYC is induced by growth factors and nutrients via PKCz^7^.

Increased MYC expression occurs not only in response to physiological stimuli, but also at pathological conditions^8^. Chronic and pronounced hyperglycemia and hyperlipidemia elevate MYC levels in beta cells, and in this case MYC induction probably promotes deleterious rather than beneficial effects on the beta cell^7,9^. Moreover, forced MYC overexpression in beta cells reduces beta cell mass and insulin secretion^10^, suggesting that MYC may contribute to the pathogenesis of diabetes by directly linking cell cycle entry attempts to beta cell dedifferentiation and apoptosis^10^. This notion is supported by the finding that sustained high levels of MYC expression in beta cells of transgenic mice have been implicated in the development of hyperglycemia in vivo^11^.

TIA1 and its homolog TIAR are RNA-binding proteins (RBPs) that play a role in the regulation of mRNA splicing, stability and translation^12,13^. One binding target for TIA1/TIAR is MYC mRNA^14,15^. Indeed, TIAR, by binding to the 3’-end of the MYC mRNA molecule, plays a crucial role in inhibiting the translation of MYC in mouse embryonic stem cells^15^. Additionally, it has been reported that also TIA1 binds MYC mRNA in neuronal cells^16^. In line with this, our recent work showed that TIA1, by binding to MYC mRNA, causes an increase in MYC protein levels and a subsequent loss of beta cell identity^17^. Interestingly, anti-MYC shRNA restored beta cell identity, supporting a role of TIA1 in MYC-mediated control of beta cell function^17^.

Furthermore, TIA1/TIAR-induced inhibition of MYC mRNA expression and translation appears to be modulated by the long non-coding RNA (lncRNA) molecule PHAROH^18^ Indeed, PHAROH increased MYC protein levels by sequestering TIAR, thereby relieving the MYC mRNA from translational inhibition^18^.

As MYC may play an important role in the pathogenesis of diabetes, the present study sought to elucidate the mechanisms by which the expression of MYC is controlled, with particular attention to the putative molecular interactions between MYC mRNA, TIA1 and PHAROH. We have also compared the expression of MYC in beta cells with that of glucagon-producing alpha cells, as alpha cells, as opposed to beta cells, retain their capacity to secrete its hormone in diabetes^19^.

## Materials and methods

### Cell culture

Mouse MIN6 cells were cultured in DMEM containing 5.6 mM glucose supplemented with 15% fetal bovine serum (Gibco, NY, USA), 2 mM L-glutamine and 50 µM beta-mercaptoethanol. Mouse α-TC1-6 cells were cultured in RPMI-1640 with 10% fetal bovine serum. Human islets were collected from the Nordic Network for Clinical Islets Transplantation at Uppsala University. CMRL-1066 medium (ICN Biomedicals, Costa Mesa, CA, USA) containing 5.6 mM glucose supplemented with 10% fetal bovine serum and 2 mM L-glutamine was employed to further culture and treatment. Above cells were cultured at 37 °C in a humidified incubator with 5% CO_2_ conditions for 1–5 days. Cell lines were used at passage numbers 50–75. Key experiments were repeated using lower passage cells, and similar results were obtained.

### Quantitative real-time PCR

Alpha-TC1-6 and MIN6 cells were incubated with 20 ng/mL human recombinant IL-1β (Peprotech, Rocky Hill, NJ, USA) + 20 ng/mL murine recombinant IFN-γ (Peprotech, Rocky Hill, NJ, USA). α-TC1-6 cells were cultured in 0.05 mM palmitate (0.5% BSA) + 22 mM glucose for 24 h. MIN6 cells were cultured with 0.4 mM palmitate (0.4% BSA) + 22 mM glucose for 24 h. Islets were incubated with 20 ng/mL human recombinant IL-1β (Peprotech, Rocky Hill, NJ, USA) + 20 ng/mL human recombinant IFN-γ (Peprotech, Rocky Hill, NJ, USA) or 0.4 mM palmitate (0.5% BSA) + 22 mM for 24 h. In separate experiments, MIN6 cells were cultured with increasing concentrations of PHAROH synthetic antisense LNA GapmeR oligonucleotides 1 and 2 (ASO 1 and ASO 2) or its scrambled control (Qiagen) for 24 h. The RNA was harvested from-α-TC1-6, MIN6 and islet cells using the Qiagen RNeasy kit (Qiagen, Hilden, Germany). The cDNA synthesis and real-time PCR was performed on a AriaDX-6 platform (Agilent) using the iTaq Universal Probes One-Step RT-qPCR kit (BioRad) with PCR primers. The relative values were then normalized to the relative amounts of GAPDH and beta-actin cDNA.

### Immunoblot analysis

MIN6 cells were preincubated with 90 nM of PHAROH synthetic antisense LNA GapmeR oligonucleotides 1 and 2 (ASO 1 and ASO 2) or its scrambled control (Qiagen) for 12 h. The cells were then cultured with cytokines or palmitate + high glucose as given above, in the presence of PHAROH ASO 1/2 or scrambled control for an additional 24 h. MIN6 cells and islets were subjected to total protein lysis using SDS sample buffer at PH 7.0, supplemented with Halt Phosphatase Inhibitor Cocktail (Thermo Fisher Scientific, Waltham, MA, USA). Immunoblotting was performed as previously described^17^ using mouse anti-MYC (1:1000, Cell Signaling), rabbit anti-TIA1/TIAR (1:1000, Santa Cruz), and mouse anti-beta-actin (1:1000, Santa Cruz) antibodies.

### RNA immunoprecipitation assay

MIN6 cells were untreated or pre-incubated for 6 h with 90 nM PHAROH ASO 1 and then another 6 h with 0.5 mM palmitate and 22 mM glucose. The cells (10^7^) were then RNA immunoprecipitated as previously described^17^.

### Statistics

The results are expressed as means ± SEM and were visualized and analyzed using Prism 10 software (Graphpad.com). For experiments involving multiple comparisons, data was analyzed using repeated measurements one-way or two-way ANOVAs, followed by Šídák’s multiple comparisons post-hoc test. In cases where only two groups were compared, a two-sided Student’s t-test was utilized.

### Data and resource availability

Sequences of PCR primers and oligonucleotides can be obtained by contacting the corresponding author.

## Results

### MYC mRNA was increased in MIN6 cells and human islets in response to cytokines or palmitate + high glucose

**Fig. 1:**
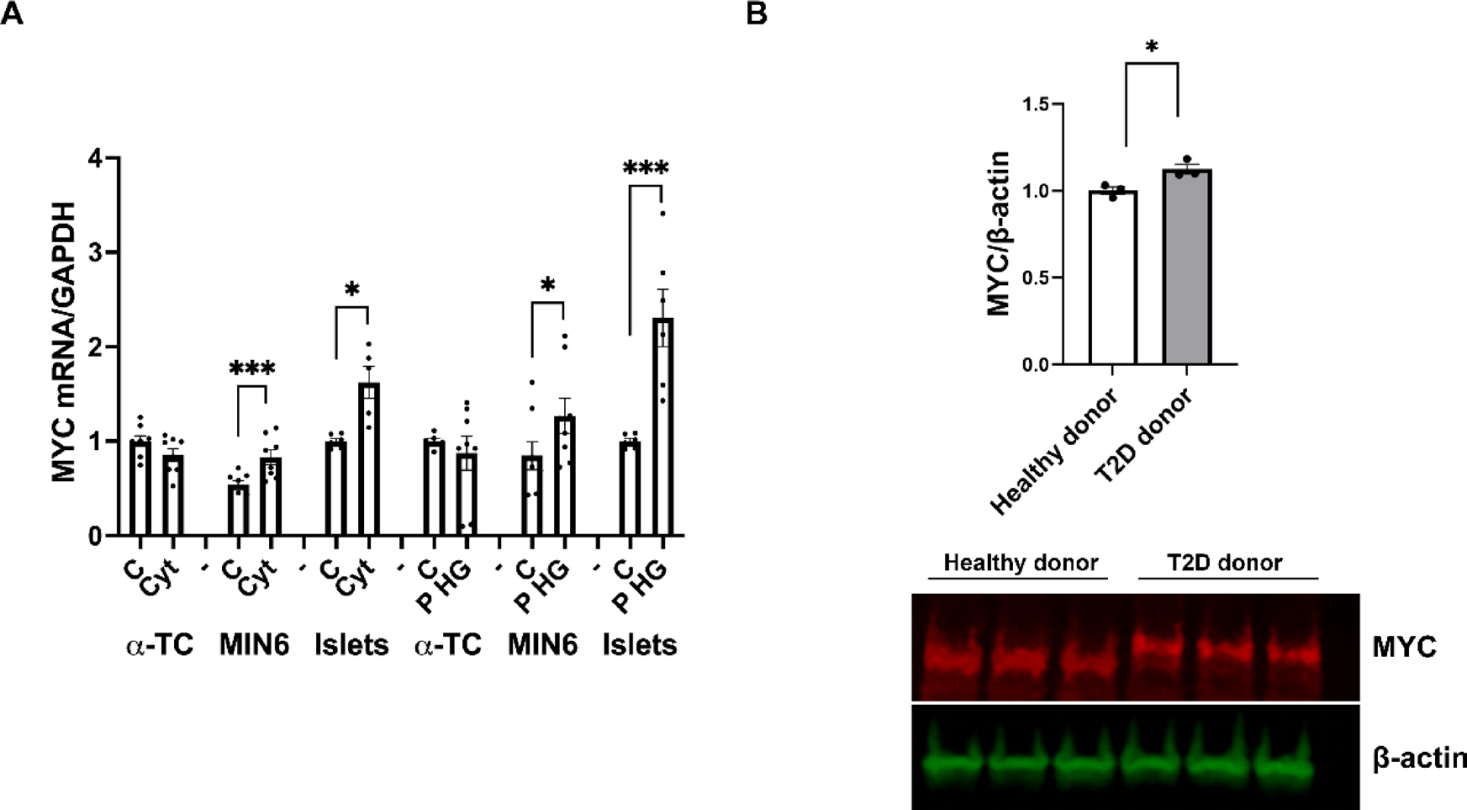
MYC mRNA levels were increased in MIN6 cells and human islets in response to cytokines or palmitate + high glucose. (**A**) MYC mRNA levels were determined in α-TC1-6 cells, MIN6 cells and human islets upon 24 h of cytokine (IL-1β + IFN-γ) or palmitate + high glucose treatment by qPCR. Results are normalized to GAPDH mRNA and to the α-TC control (C), and are means ± SEM for 5–8 experiments. * *P* < 0.05 and *** *P* < 0.001 using two-way repeated measurement ANOVA and Šídák’s post hoc test. (**B**) MYC protein levels were determined in human islets from non-diabetic and T2DM organ donors and quantification was performed using Image J software. * *P* < 0.05 using Student’s t-test (*n* = 3).

The overexpression of MYC has been demonstrated to inhibit insulin gene transcription and glucose-stimulated insulin secretion in pancreatic beta cells, highlighting its involvement in beta-cell dysfunction and the pathogenesis of diabetes^7,9^. In the present study, MYC expression in MIN6 cells and human islets was upregulated by treatment with the proinflammatory cytokines IL-1β and IFN-γ or palmitate + high glucose (Fig. 1A). Conversely, no significant changes were observed in α-TC1-6 cells, suggesting that inflammatory and glucolipotoxicity treatments selectively influence MYC expression in islet beta cells, rather than in alpha cells. We also studied MYC protein levels in human islets from control donors and T2D donors, and found that MYC was increased in the T2D islets (Fig. 1B).

### PHAROH lncRNA was increased in MIN6 cells in response to cytokines or palmitate + high glucose

**Fig. 2:**
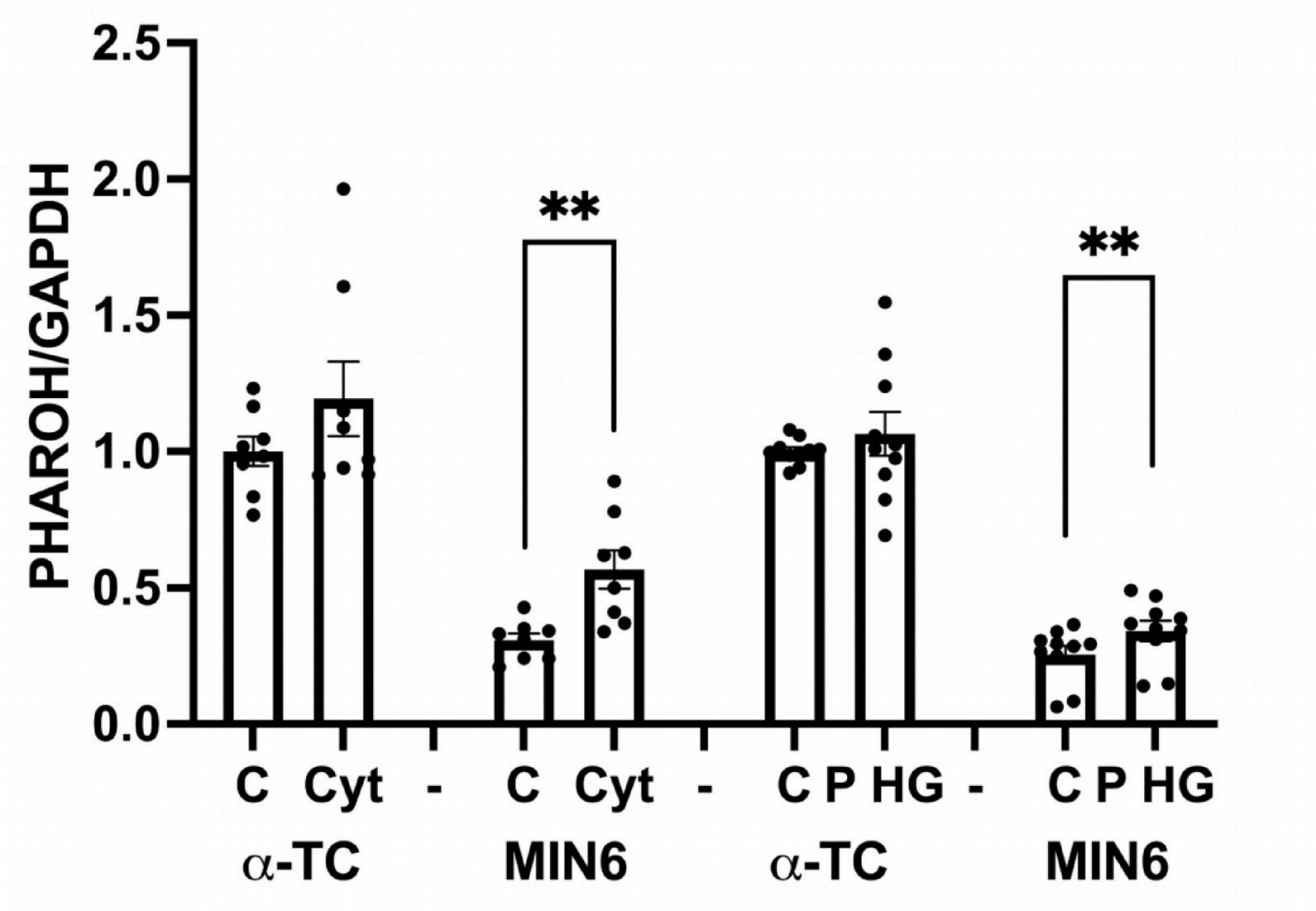
PHAROH lncRNA levels were increased in MIN6 cells in response to cytokines or palmitate + high glucose. PHAROH lncRNA levels were determined in α-TC1-6 and MIN6 cells following treatment with cytokines or palmitate + high glucose by qPCR analysis. Results are normalized to GAPDH mRNA and to the α-TC control (C), and are means ± SEM for 8–10 experiments. ** *P* < 0.01 using two-way repeated measurement ANOVA and Student’s t-test as post hoc test.

A previous study reported that PHAROH lncRNA modulates MYC mRNA translation in murine hepatocellular carcinoma by sequestering the RNA-binding protein TIAR, a homologue to TIA1^18^. Given that no homo sapiens ortholog to murine PHAROH has been described, its expression was only assessed in murine α-TC1-6 and MIN6 cells, and not in human islets. Similarly to the MYC mRNA expression pattern (Fig. 1A), PHAROH expression was also upregulated following cytokine or glucolipotoxicity treatment in MIN6 cells, but not in α-TC1-6 cells, suggesting that changes in PHAROH expression may control MYC expression in beta cells (Fig. 2).

### Targeting PHAROH with antisense LNA GapmeR ASO 1 promotes increased MYC mRNA levels

**Fig. 3:**
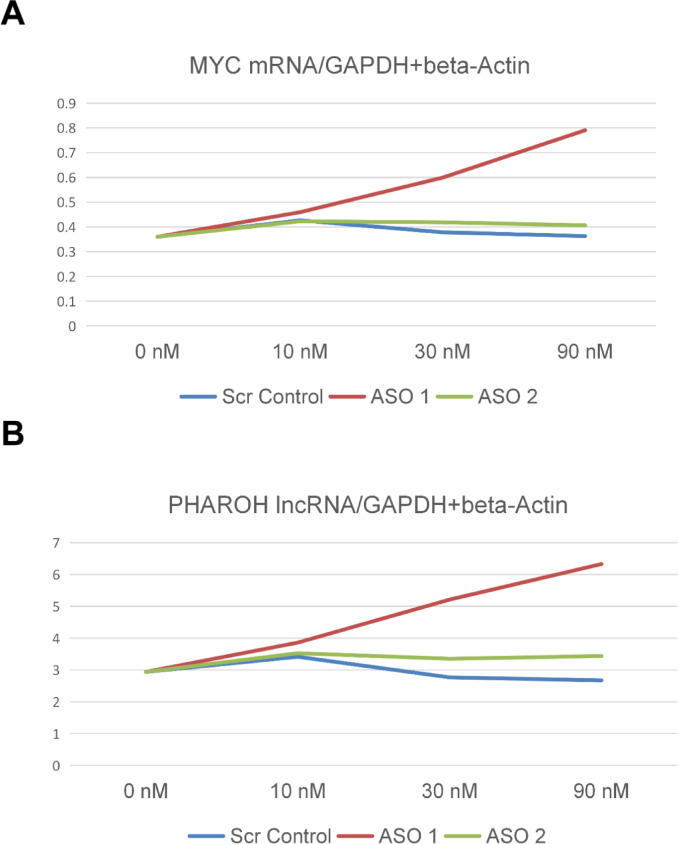
PHAROH ASO 1 increased PHAROH lncRNA and MYC mRNA levels. MIN6 cells were incubated for 24 h with increasing concentration of Scrambled neg control (Scr Control), PHAROH ASOs 1 and 2, which was followed by qPCR quantification of PHAROH lncRNA and MYC mRNA levels. Results were normalized to the average of GAPDH and beta-actin mRNA levels.

To study whether PHAROH lncRNA participates in cytokine or palmitate + high glucose-induced MYC expression, we designed two antisense LNA GapmeR molecules, one targeting the 3’ region (PHAROH ASO 1) and one targeting the 5’ region (PHAROH ASO 2). These antisense molecules are known to be taken up spontaneously during cell culture and to reduce expression/activity of the target RNA. MIN6 cells were incubated for 24 h with increasing concentrations of the PHAROH ASOs. PHAROH lncRNA and MYC mRNA levels were then analyzed using a scrambled LNA GapmeR as control. We observed that PHAROH ASO 1 increased MYC mRNA levels dose dependently (Fig. 3A). Surprisingly, PHAROH ASO 1 increased also PHAROH lncRNA levels (Fig. 3B), suggesting that the inhibitor promoted increased stability, rather than the expected loss of stability. PHAROH ASO 2 affected neither PHAROH nor MYC expression (Fig. 3A + B). Based on these findings, further experimentation was performed with PHAROH ASO 1 assuming that it functioned as an activator, rather than an inhibitor.

### MYC protein levels were increased by PHAROH ASO 1 in MIN6 cells

**Fig. 4:**
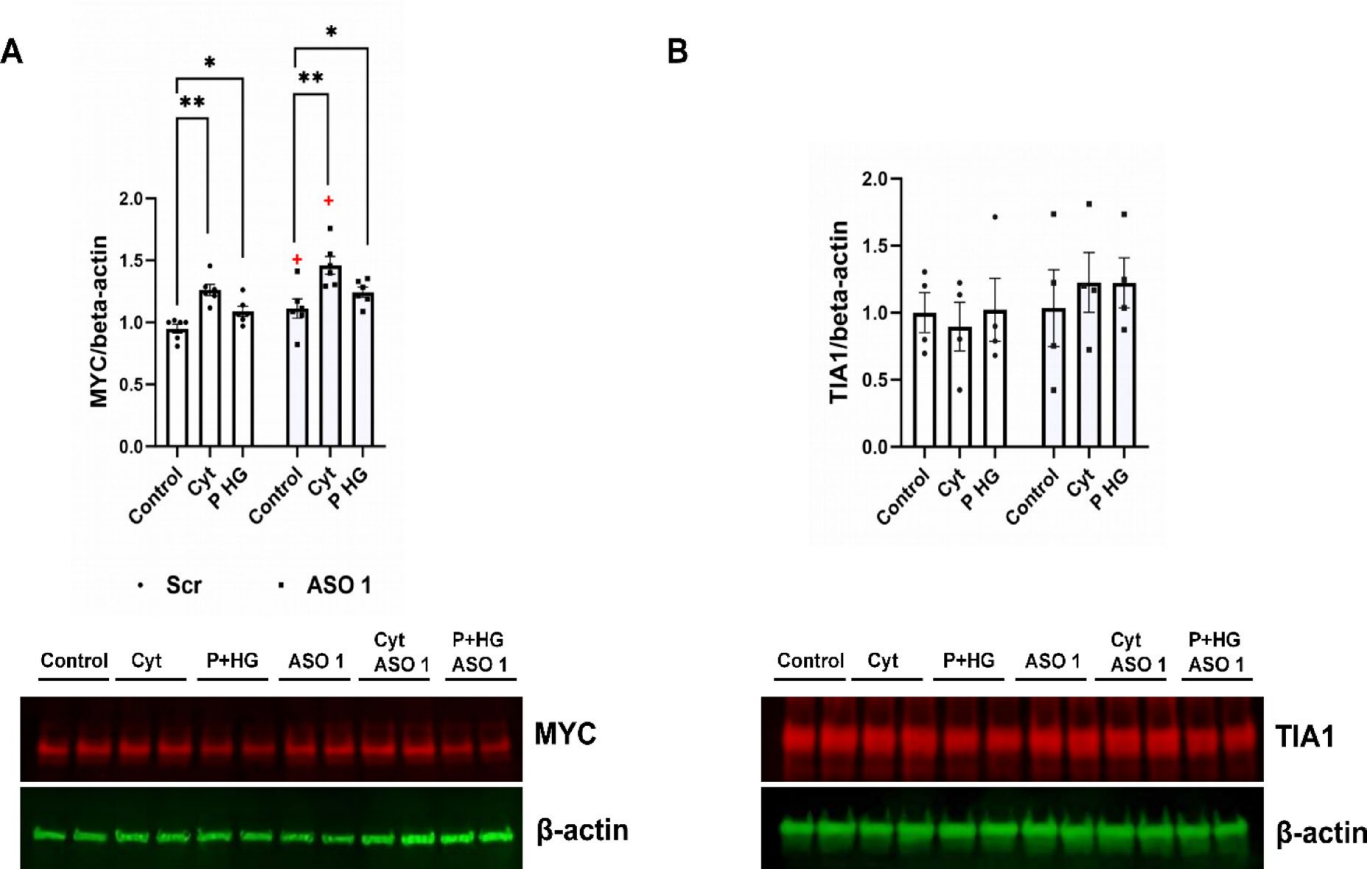
MIN6 cell MYC protein levels were increased by cytokines, palmitate + high glucose and PHAROH ASO 1*.* MIN6 cells were preincubated with 90 nM PHAROH ASO 1 for 12 h and then exposed to cytokines or palmitate plus high glucose for an additional 24 h in the presence or absence of PHAROH ASO 1. MYC protein (**A**) and TIA1 protein (**B**) levels were analyzed by immunoblotting. MYC levels were normalized to beta-actin levels. Results are means ± SEM for 6 (panel A) and 4 (panel B) experiments. * *P* < 0.05 and *** *P* < 0.001 using two-way repeated measurement ANOVA and Šídák’s post hoc test. + denotes *P* < 0.05 using two-way repeated measurement ANOVA and Šídák's as post hoc test when comparing vs. corresponding Scr control.

Based on previous reports showing that PHAROH regulates MYC via competitive interaction with TIA1/TIAR^18^, we hypothesized that PHAROH upregulation induced by PHAROH ASO 1 would sequester TIA1 away from MYC mRNA. This would then cause an increase in MYC protein levels, in a manner consistent with the changes observed following cytokine or palmitate plus high-glucose treatment (Figs. 2 and 3). Therefore, we analyzed whether MYC protein levels were augmented in response to the different treatments. MYC protein levels were increased in MIN6 cells by both cytokines and palmitate + high glucose (Fig. 4A). Using a concentration of 90 nM of PHAROH ASO 1, we observed that MIN6 cell MYC protein levels were further increased with/without the presence of cytokines (Fig. 4A). In palmitate + high glucose treated MIN6 cells, the P-value was 0.058. In contrast, TIA1 protein levels remained unaffected by either cytokine or palmitate + high glucose treatments, or by PHAROH ASO 1 exposure (Fig. 4B), indicating that changes in MYC protein levels were not dependent on alterations in TIA1 protein levels. These results suggest that increases in PHAROH lncRNA may participate in augmenting MYC protein levels.

### MYC mRNA binding to TIA1 was decreased in response to palmitate + high glucose and PHAROH ASO 1

**Fig. 5:**
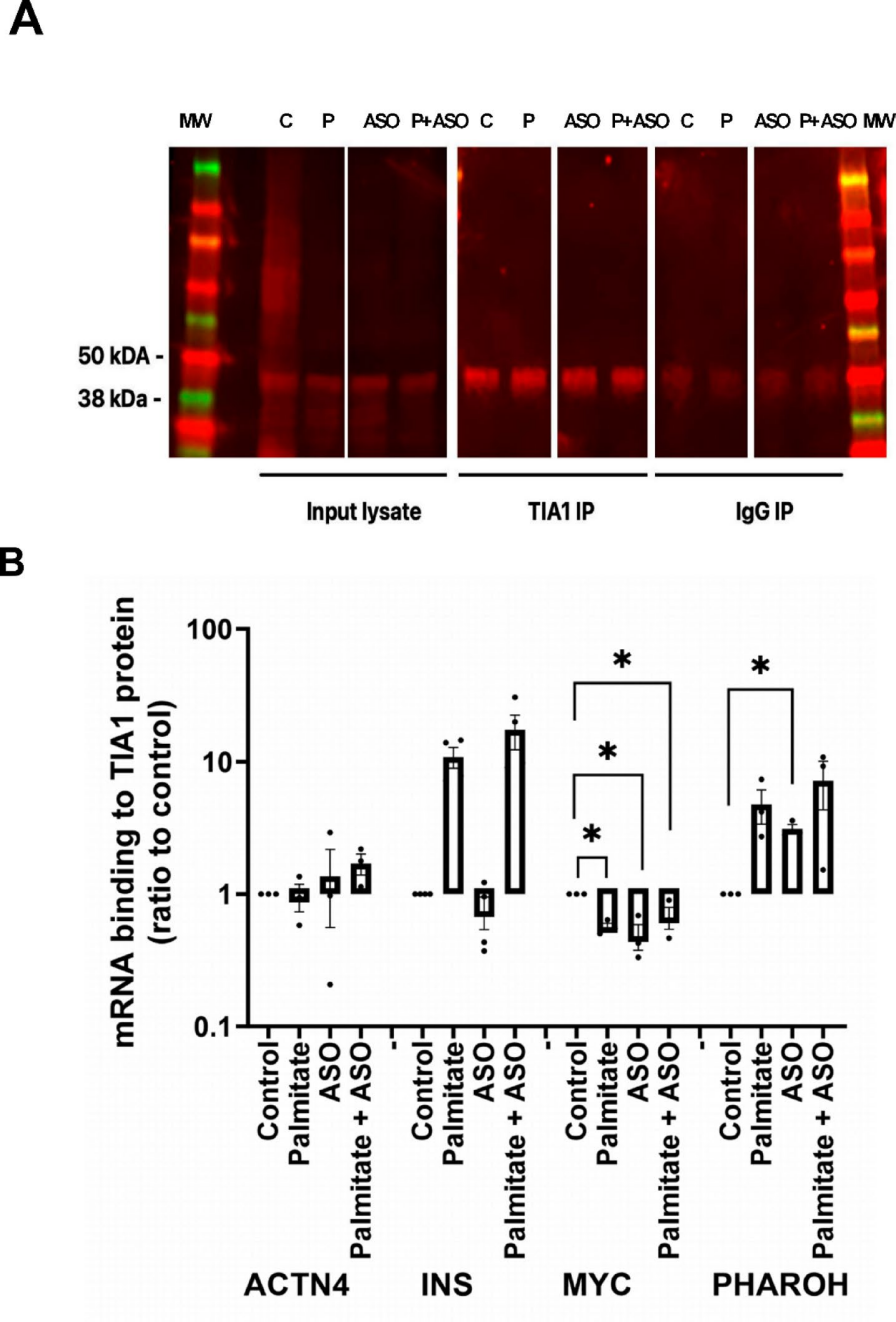
PHAROH ASO 1 decreases binding of MYC mRNA to TIA1, and increases binding of PHAROH to TIA1. MIN6 cells were preincubated with 90 nM PHAROH ASO 1 for 6 h, followed by exposure to palmitate plus high glucose for another 6 h. TIA1 was then immunoprecipitated and associated RNAs were quantified by qPCR*.* (**A**) TIA1 and IgG protein in input lysate and IP of MIN6 cells were immunoprecipitated and determined by Western blot. (**B**) Levels of ACTN4, INS, MYC and PHAROH RNA were quantified using qPCR. Results are means ± SEM for 3 experiments. * *P* < 0.05 and ** *P* < 0.01 using one-way repeated measurement ANOVA and Šídák’s post hoc test.

As we observed increased MYC levels in MIN6 cells exposed to cytokines or palmitate + high glucose, possibly caused by increased PHAROH levels that prevent MYC mRNA binding to TIA1, we next investigated whether mRNA binding to TIA1 was affected by stress treatments and PHAROH ASO 1. TIA1 was immunoprecipitated from MIN6 cell lysates, with IgG used as a control. Input lysates and immunoprecipitates were analyzed by Western blot (Fig. 5A). The binding of MYC mRNA to the TIA1 protein was reduced in the presence of both palmitate + high glucose and PHAROH ASO 1 (Fig. 5B). On the other hand, PHAROH binding to TIA1 was increased by the same treatments, suggesting that increased PHAROH levels resulted in a replacement of MYC mRNA with PHAROH. Insulin mRNA binding to TIA1 was strongly increased by palmitate + high glucose, but was not affected by the PHAROH ASO 1. This indicates that insulin mRNA during stress is relocated from active translation to TIA1-containing stress granules, and that PHAROH does not compete with this process. Binding of TIA1 to ACTN4 mRNA, a strong TIA1 binding target in neuronal cells^16^, was not affected by palmitate + high glucose, or by PHAROH ASO 1. Thus, the TIA1/ACTN4 mRNA association is neither affected by PHAROH levels, nor by metabolic stress.

## Discussion

The control of MYC expression is a highly complex process involving gene amplification-, epigenetic-, transcriptional- and posttranscriptional mechanisms^19^. In beta cells, transcriptional control of the MYC gene has not been well studied, but specific posttranslational events have been reported to enhance MYC Ser62 phosphorylation and increased MYC protein stability^8^. In addition, we recently reported that human EndoC-betaH1 cell MYC protein levels are controlled by the binding of TIA1 to MYC mRNA^17^. Furthermore, studies performed in different types of cancer cells report that MYC expression is controlled by interactions between the MYC mRNA molecule and specific long non-coding RNAs (lncRNA) that affect MYC mRNA transcription, stability and translation^18,20–22^. As dysregulation of MYC has been linked to beta cell dysfunction in diabetes, we presently assessed the possibility that MYC levels in beta cells are controlled via a similar lncRNA-dependent mechanism involving PHAROH, and that the MYC mRNA-binding protein TIA1 participates in this interaction. Indeed, our results suggest that TIA1, at basal conditions (5.6 mM glucose without cytokines or palmitate), by binding to MYC mRNA reduces MYC protein levels. This finding resembles those observed in other cell types^14,15^. During stress, however, PHAROH levels increased, which made TIA1 relocate from MYC mRNA to PHAROH, resulting in increased MYC mRNA and protein levels. As the effect of stress was mimicked by PHAROH ASO 1, an oligonucleotide that increased PHAROH levels, it is likely that PHAROH plays a causative role, and not only a correlative role, in this chain of events. That PHAROH ASO 1 increased PHAROH is an unexpected and not easily explained finding, but it can be speculated that the PHAROH ASO 1, by binding to its site on PHAROH, blocks a specific high-affinity RNase binding site, and that this leads to a paradoxical increase in stability^23–26^.

While our study implicates PHAROH in modulating MYC mRNA post-transcriptional regulation via competitive binding to TIA1, it does not appear to exert effects on TIA1 interactions with other mRNA molecules. Specifically, insulin mRNA binding to TIA1 was markedly increased under glucolipotoxic conditions, demonstrating that metabolic stress promotes sequestration of insulin transcripts into TIA1-containing stress granules, thereby reducing insulin biosynthesis and secretion. However, PHAROH ASO 1 had no detectable impact on this process, suggesting that PHAROH does not interfere with TIA1-mediated inhibition of insulin expression.

ACTN4 is a cytoskeletal regulator and a well-established TIA1 target in neuronal cells^16^. The unaffected stability of TIA1–ACTN4 mRNA binding under both stress and PHAROH ASO 1 conditions suggests that PHAROH does not displace high-affinity canonical TIA1 targets, at least in non-neuronal contexts. The lack of change in ACTN4 binding suggests that TIA1 binding mRNA targets can be divided into compartmentalized pools—some engaged in core stress granule assembly, some dynamically regulated by PHAROH lncRNAs, and some not.

Islet alpha cells play a crucial role in the production and release of glucagon, and are known to be less sensitive than beta cells to metabolic stress^27^. Interestingly, alpha cells, as opposed to beta cells, did not increase their MYC and PHAROH expression in response to cytokine exposure or glucolipotoxicity. The reason for this is not clear, but the inability of alpha cells to upregulate PHAROH and MYC, in response to glucolipotoxic conditions, might contribute to their higher resistance to metabolic stress^27^, as MYC upregulation promotes, rather than ameliorates, beta cell dysfunction during stress^7,9^. Other factors that appear to mediate alpha cell resistance to stress are increased BCL2L1 and HSPA5 levels and decreased CHOP levels^28^.

In summary, we propose that stress-induced upregulation of PHAROH enables beta cells to override TIA1-mediated translational repression of MYC. This seems to occur without affecting other transcripts (insulin mRNA), thereby selectively amplifying MYC expression in the context of inflammatory and metabolic insults. Thus, PHAROH provides a novel layer of post-transcriptional control of MYC expression, with potential implications for understanding how beta cells balance adaptation versus dysfunction under diabetogenic stress. It may be that this mechanism contributes both to the beta cell’s sensitivity to stress, and to its compensatory proliferation. Future work should aim to identify human orthologues of PHAROH, and/or other lncRNA molecules involved in the regulation of MYC expression, and to explore therapeutic opportunities to modulate this axis in diabetes.

A major limitation of this study is that the expected reduction of PHAROH levels was not achieved using PHAROH targeting ASOs. Thus, we have not been able to study effects of PHAROH knock-down, but instead only PHAROH upregulation. Furthermore, we have also not studied whether PHAROH-induced MYC activity affected beta cell identity and function. Finally, most mechanistic experiments were performed in cell line models, and further studies using primary islet cells and in vivo systems will be required to confirm the physiological relevance of the PHAROH–TIA1–MYC regulatory axis.

## Supplementary Information

Below is the link to the electronic supplementary material.

Supplementary Material 1

Supplementary Material 2

## Data Availability

The datasets generated and/or analyzed during the current study are available from the corresponding author upon reasonable request.

## Abbreviations

ASO: Antisense oligonucleotide
BSA: Bovine serum albumin
lncRNA: Long non-coding RNA
RBP: RNA-binding protein
T1D: Type 1 diabetes
T2D: Type 2 diabetes
TIA1: T-cell intracellular antigen 1
TIAR: T-cell intracellular antigen 1-related
