# Supplementary material for: The lncRNA PHAROH augments MYC mRNA levels in insulin-producing cells by counteracting TIA1-induced MYC silencing
Source: Sci Rep. 2026 Aug 18;16:25916. doi: 10.1038/s41598-026-65911-9 (PMC13486764; doi:10.1038/s41598-026-65911-9)

Fig 1B MYC

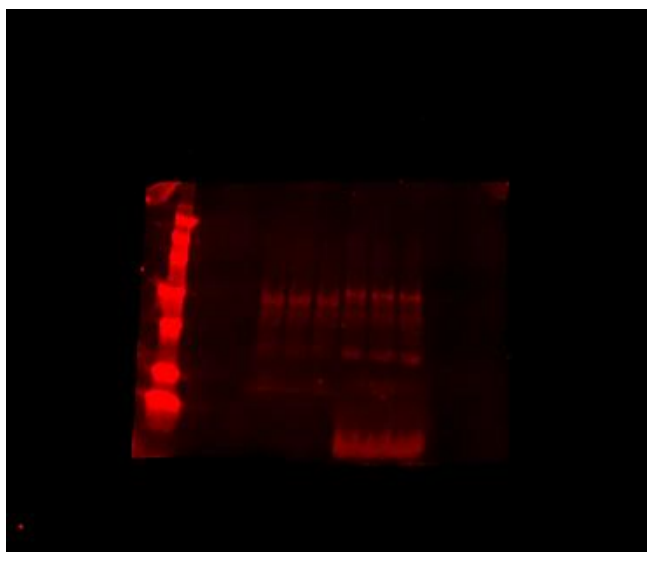

Fig 1B actin

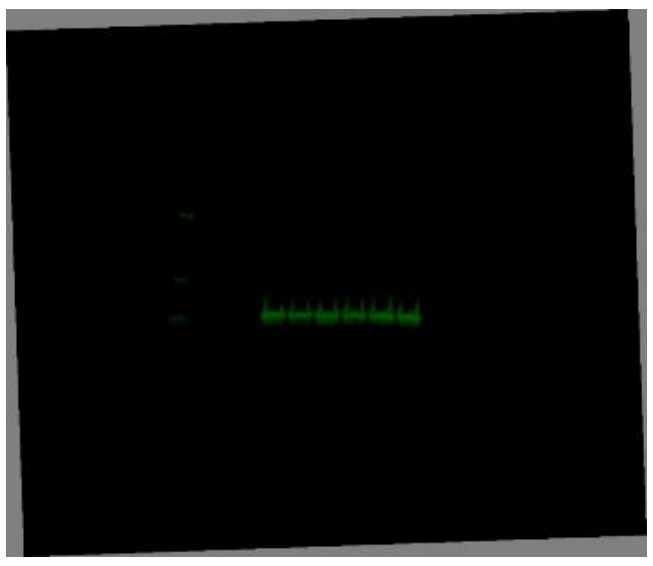

Fig 4A MYC

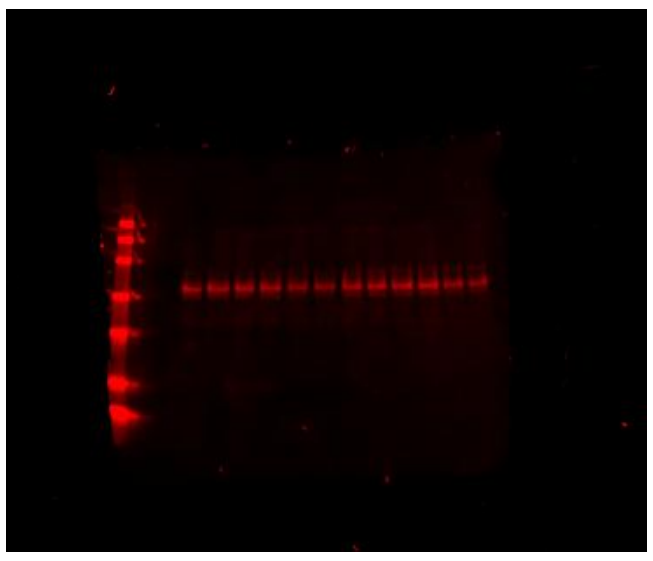

Fig 4A actin

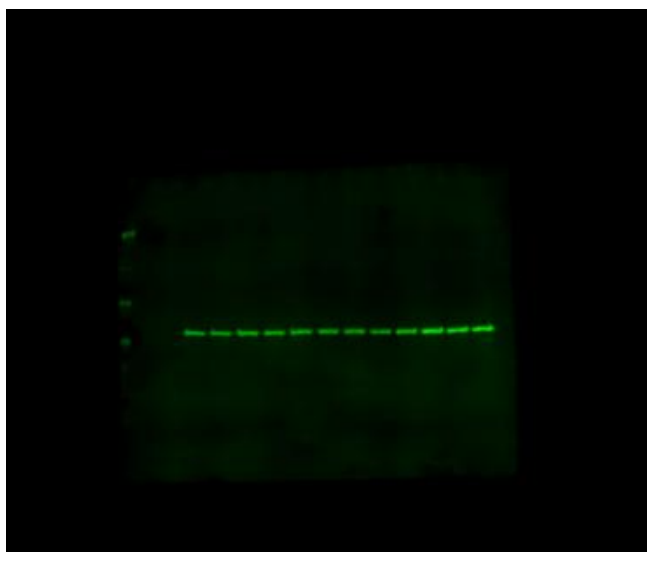

Figure 4B TIA1

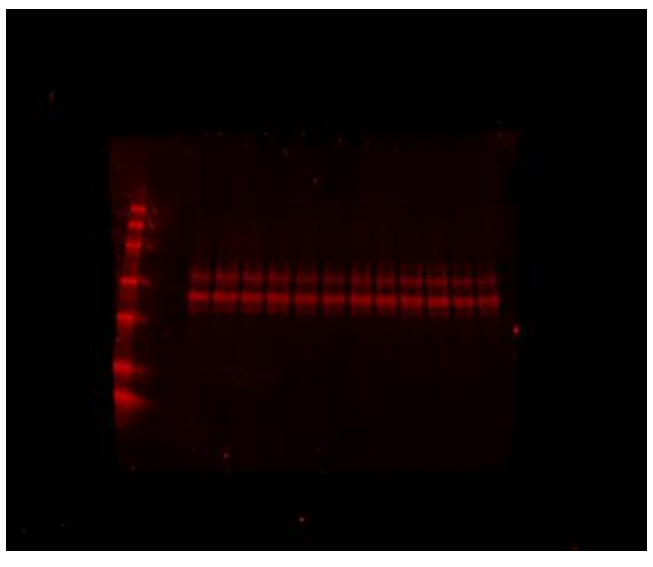

Fig 4B actin

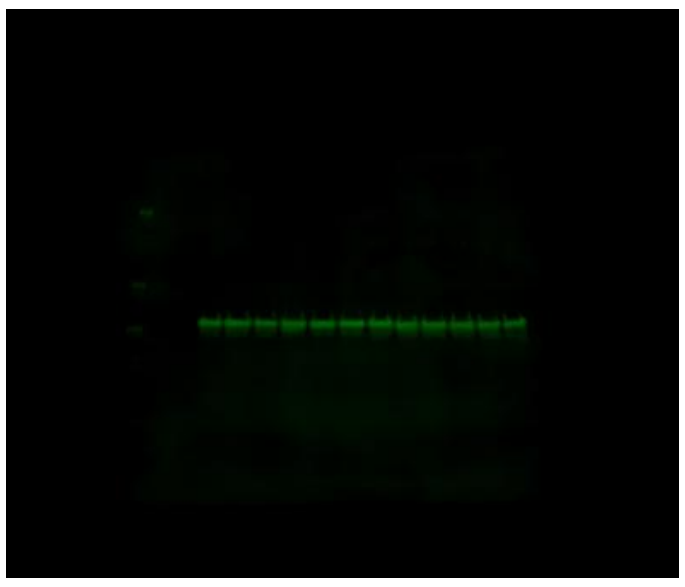

Fig 5A (input lysate , lane 2, 3, 4, 5)

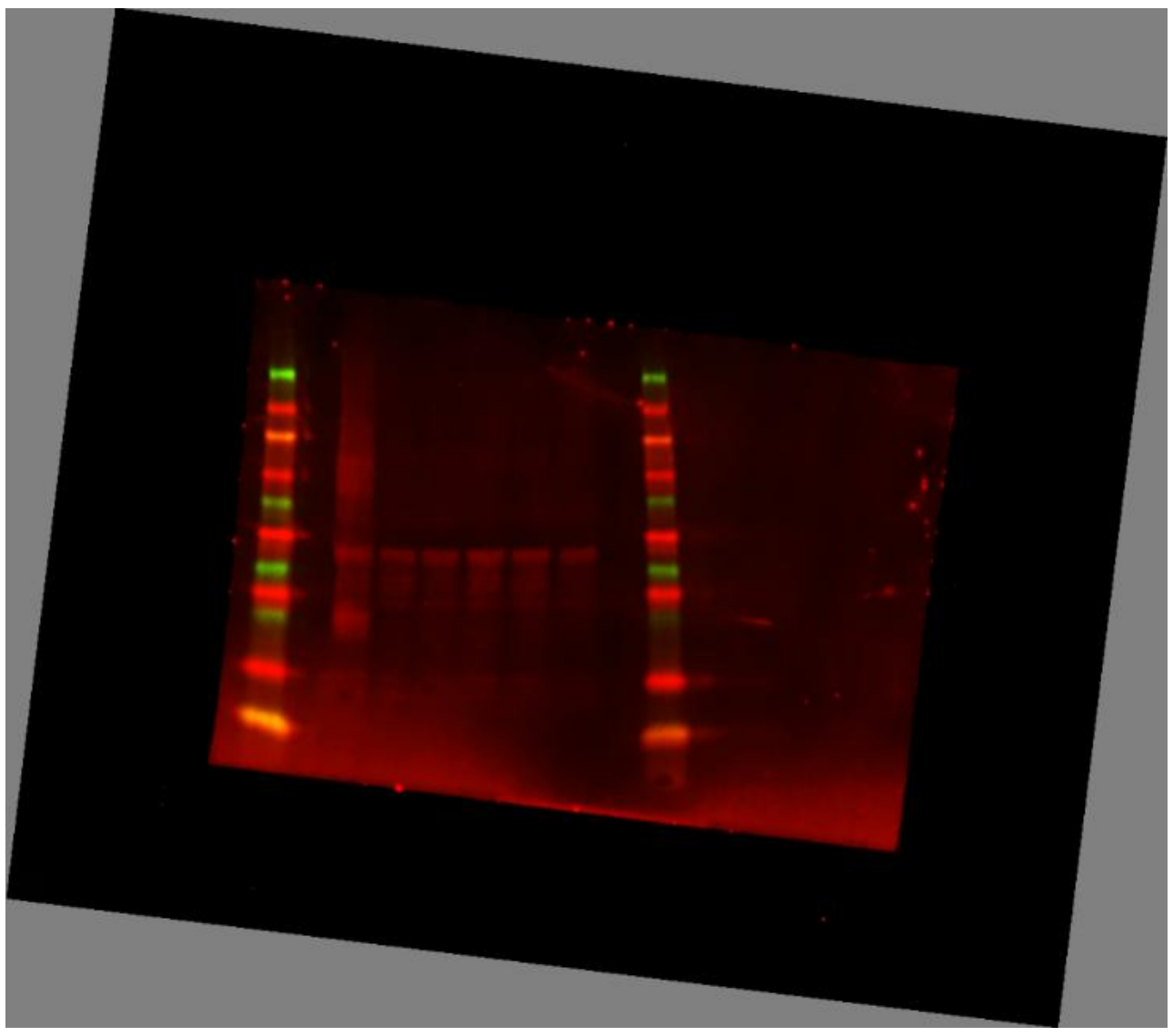

Fig 5A (TIA1 IP: from right to left lane 1,2, 5, 6)
(IgG IP: from right to left lane 7,8 ,11,12)

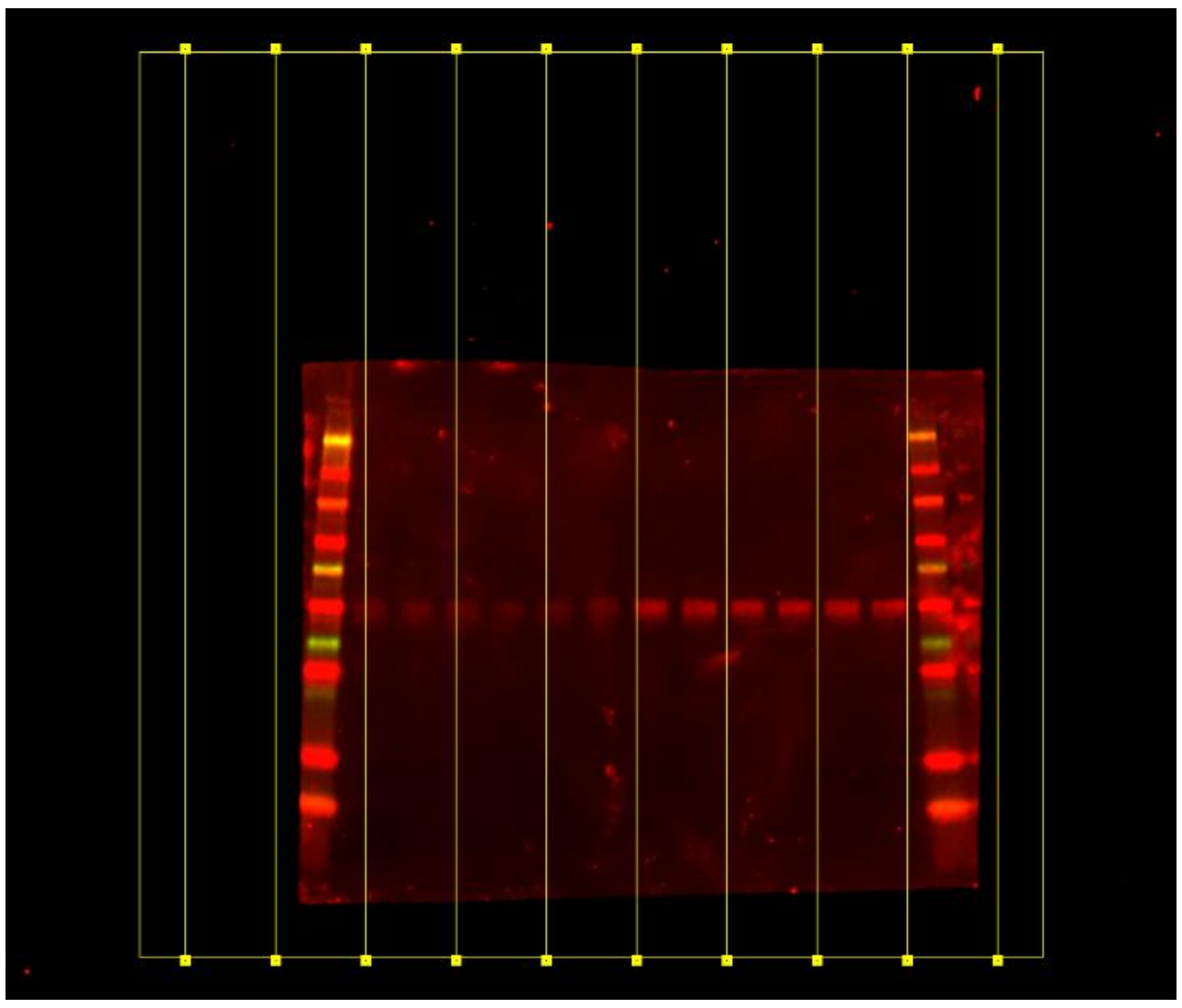

Supplement: Supplementary file 1 [file 41598_2026_65911_MOESM1_ESM.pdf]
