# Supplementary material for: The lncRNA PHAROH augments MYC mRNA levels in insulin-producing cells by counteracting TIA1-induced MYC silencing
Source: Sci Rep. 2026 Aug 18;16:25916. doi: 10.1038/s41598-026-65911-9 (PMC13486764; doi:10.1038/s41598-026-65911-9)
Supplement: Supplementary file 2 [file 41598_2026_65911_MOESM2_ESM.pdf]

	Forward (5'-3')	Reverse (5'-3')
Mus Insulin	GGCTCTCTACCTGGTGTGTGG	CCAGCTGGTAGAGGGAGCAG
Mus ACTN4	ATGGTGGACTACCACGCAG	CAGCCTTCCGAAGATGAGAGT
Mus and Homo MYC	GAGGTGGAGCAGACGCTGT	CCAGGACTGTATGTGGAGCGG
Mus PHAROC	GGGGCCAAGAAGATGACACG	GGACGCATGTGGAGGTCAGA
Mus and Homo GAPDH	GTCTCCTCTGACTTCAACAGCG	ACCACCCTGTTGCTGTAGCCAA

Suppl. Table 1
Primers used for qPCR
